# Microgeographic local adaptation and ecotype distributions: The role of selective processes on early life‐history traits in sympatric, ecologically divergent *Symphonia* populations

**DOI:** 10.1002/ece3.6731

**Published:** 2020-09-17

**Authors:** Niklas Tysklind, Marie‐Pierre Etienne, Caroline Scotti‐Saintagne, Alexandra Tinaut, Maxime Casalis, Valerie Troispoux, Saint‐Omer Cazal, Louise Brousseau, Bruno Ferry, Ivan Scotti

**Affiliations:** ^1^ INRAE UMR0745 EcoFoG AgroParisTech Cirad CNRS Université des Antilles Université de Guyane Kourou Cedex France; ^2^ CNRS IRMAR ‐ UMR 6625 Univ Rennes Rennes France; ^3^ INRAE UR629 Ecologie des Forêts Méditerranéennes (URFM) Avignon France; ^4^ Université de Guyane UMR0745 EcoFoG INRAE AgroParisTech Cirad CNRS Université des Antilles Kourou Cedex France; ^5^ AgroParisTech INRAE UMR SILVA Université de Lorraine Nancy France; ^6^ Present address: UMR AMAP IRD Cirad CNRS INRAE Université Montpellier Montpellier France

**Keywords:** determinants of plant community diversity and structure, evolutionary ecology, landscape ecology, local adaptation, Neotropical forest, plant development and life‐history traits, reciprocal transplantation experiments, *Symphonia*

## Abstract

Trees are characterized by the large number of seeds they produce. Although most of those seeds will never germinate, plenty will. Of those which germinate, many die young, and eventually, only a minute fraction will grow to adult stage and reproduce. Is this just a random process? Do variations in germination and survival at very young stages rely on variations in adaptations to microgeographic heterogeneity? and do these processes matter at all in determining tree species distribution and abundance?

We have studied these questions with the Neotropical *Symphonia* tree species. In the Guiana shield, *Symphonia* are represented by at least two sympatric taxa or ecotypes, *Symphonia globulifera* found almost exclusively in bottomlands, and a yet undescribed more generalist taxon/ecotype, *Symphonia sp1*. A reciprocal transplantation experiment (510 seeds, 16 conditions) was set up and followed over the course of 6 years to evaluate the survival and performance of individuals from different ecotypes and provenances.

Germination, survival, growth, and herbivory showed signs of local adaptation, with some combinations of ecotypes and provenances growing faster and surviving better in their own habitat or provenance region. *S. globulifera* was strongly penalized when planted outside its home habitat but showed the fastest growth rates when planted in its home habitat, suggesting it is a specialist of a high‐risk high‐gain strategy. Conversely, *S. sp1* behaved as a generalist, performing well in a variety of environments.

The differential performance of seeds and seedlings in the different habitats matches the known distribution of both ecotypes, indicating that environmental filtering at the very early stages can be a key determinant of tree species distributions, even at the microgeographic level and among very closely related taxa. Furthermore, such differential performance also contributes to explain, in part, the maintenance of the different *Symphonia* ecotypes living in intimate sympatry despite occasional gene flow.

## Graphical abstract

A reciprocal transplantation experiment (510 seeds, 16 conditions) was setup and followed over the course of 6 years to evaluate the survival and performance of individuals from two different *Symphonia* ecotypes and provenances. The measured traits showed signs of local adaptation, with some combinations of ecotypes and provenances growing faster and surviving better in their own habitat or provenance region. The differential performance of seeds and seedlings in the different habitats matches the known distribution of both ecotypes, indicating that environmental filtering at the very early stages can be a key determinant of tree species distributions, even at the microgeographic level and among very closely related taxa.

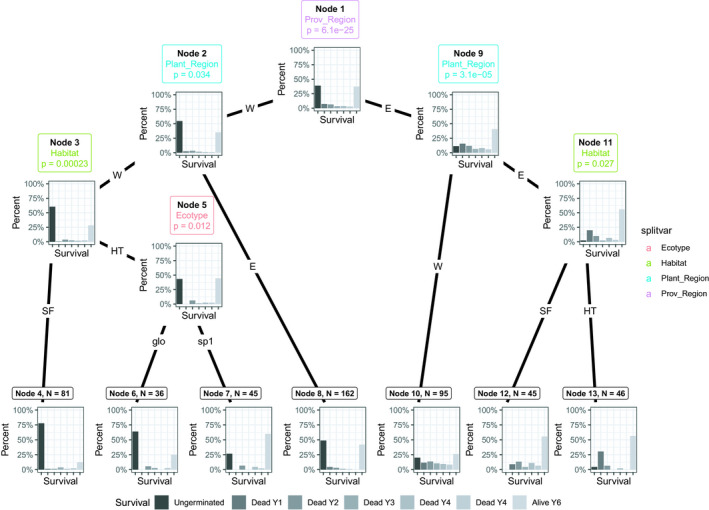

## 1 INTRODUCTION

Trees may produce millions of seeds over their lifespan (Moles, Leishman, Falster, & Westoby, 2004), yet the vast majority of those seeds will never become adults. Most of them will disappear in early life stages, when mortality is high (Petit & Hampe, 2006; Valen, 1975)⁠, through herbivory, disease, lack of resources like water (Slot & Poorter, 2007),⁠ and maladaptation to their environment (Donohue, Rubio de Casas, Burghardt, Kovach, & Willis, 2010). Such high mortality rates should provide ample opportunity for the action of natural selection on genetic diversity (Donohue et al., 2010; Petit & Hampe, 2006; Postma & Ågren, 2016). Even though one must expect that any given seedling has much higher chances to die than to survive, identifying signals of adaptation to local microenvironmental conditions in young seedlings is paramount to understand species and phenotype distribution patterns. However, the causal links between environmental heterogeneity, spatial distribution of species and phenotypes, and local adaptation remain elusive in trees, mainly due to the lack of long‐term studies, low statistical power, and insufficient understanding of the environmental factors determining local adaptation.

Local genetic differentiation and its adaptive significance are widely recognized in plants, and evidence of microgeographic adaptive processes is accumulating for trees (Barton, Jones, Edwards, Shiels, & Knight, 2020; Brousseau, Bonal, Cigna, & Scotti, 2013; Brousseau et al., 2018; Brousseau, Foll, Scotti‐Saintagne, & Scotti, 2015; Carsjens et al., 2014; Pluess et al., 2016; Ramírez‐Valiente et al., 2009; Rellstab et al., 2016; Wright, 2007). Differences in seedling performances along ecological gradients are typically interpreted as underlying observed interspecific differences in the distribution of mature trees. The study of seedling survival and growth is a therefore straightforward way to make inferences on performance differences among tree species and populations (Baraloto, Goldberg, & Bonal, 2005)⁠.

Reciprocal transplant experiments (RTE), whereby seeds or seedlings are translocated between sites in the field and grow in the same conditions as natural regeneration, are an elegant way to test the hypothesis of a link between habitat variation and species or phenotype distribution, because they allow observing performance variance directly across an array of environmental factors (Morris et al., 2007). These differences in performance components (i.e., survival, growth, and/or reproduction) between populations in different environments can be interpreted directly in terms of adaptation, based on straightforward theoretical expectations: Local adaptation is found when local populations perform better than transplanted ones (the “local vs. foreigner” condition) and any given population performs better in its own provenance than elsewhere (the “home vs. away” condition) (Kawecki & Ebert, 2004). Local adaptation can be further nuanced depending on the generality of patterns across the system: If “local” individuals always outperform “foreign” individuals for all populations and conditions, then genetic trade‐offs are orchestrating the patterns of local adaptation; if, however, some populations perform better at “home,” but are not penalized elsewhere, then conditional neutrality may underpin the local adaptation. The two processes are not mutually exclusive and may coexist for different traits within a given system (Wadgymar et al., 2017).

**FIGURE 1:**
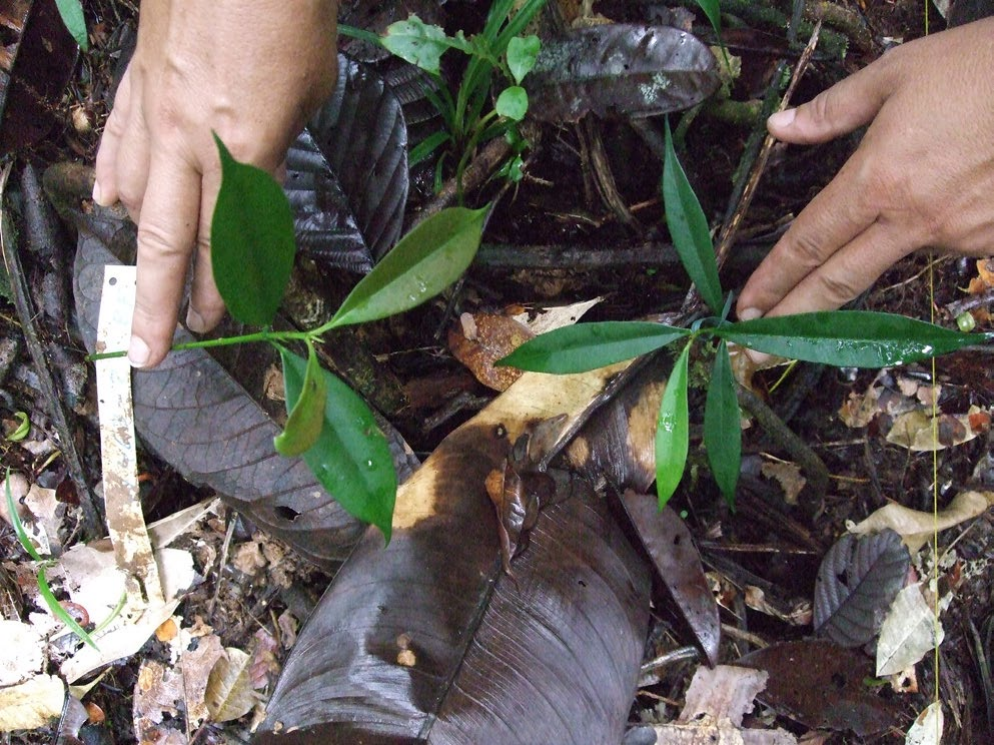
Picture of *Symphonia* seedlings belonging to *S. globulifera* (left) and *S. sp1* (right) growing next to each other in the reciprocal transplantation gardens. Credit: Maxime Casalis

Lowland Neotropical forest, such as those in French Guiana, show a highly variable and complex mosaic of microhabitats linked to variations in topography and soil characteristics. Differences in water drainage have been long identified as a main ecological factor driving the tree community composition on the Guiana shield (Barthes, 1991; Sabatier et al., 1997; ter Steege, Jetteer, Polak, & Werger, 1993), allowing to position the species along a gradient of tolerance to prolonged water saturation of soil porosity and a gradient of tolerance to temporary water saturation (Pélissier, Dray, & Sabatier, 2002). All these studies show that the most striking variations in tree species distribution at local scale result from the widespread gradient between seasonally flooded (SF) habitats along streams and all other surrounding habitats on slopes and hilltops (HT). These studies also showed that congeneric species often display opposite niche preferences across such gradients, as was pointed out by Allié et al. (2015). A typical example is provided by the genus *Symphonia* of African origin, with the species *S. globulifera,* widespread across the Neotropics, and a morphotaxon of yet undetermined status, currently identified as *S. sp1* (Baraloto, Morneau, Bonal, Blanc, & Ferry, 2007; Molino & Sabatier, 2001; Schmitt, Hérault, et al., 2020), which is known to occur in the Guiana shield. Adults of the two taxa, which for the purposes of the present study are conservatively referred to as “ecotypes,” are found in sympatry, often with intermingled crowns, but are environmentally segregated, with *S. globulifera* being strongly linked to SF habitats, while *S. sp1* is found on both HT and SF ( Allié et al., 2015; Schmitt et al., 2020). The two ecotypes are differentiated by their leaf traits (Figure 1), overall size of their leaves, flowers, and fruits, the texture of their bark, the presence of pneumatophores or prop roots (Baraloto et al., 2007; Schmitt, Hérault, et al., 2020), as well as differences in maximum diameter at breast height and growth rates (Hérault et al., 2011). Whether these morphological differences are the product of environmentally driven phenotypic plasticity or genetically determined is not yet known. The system constitutes an extreme case of microgeographic differentiation and, potentially, adaptation (Richardson, Urban, Bolnick, & Skelly, 2014), as trees of the two ecotypes are distributed in mosaic patches smaller than the pollen dispersal potential (20–50 m) (Degen, Bandou, & Caron, 2004). The two ecotypes are genetically differentiated based on nuclear microsatellites (*F*_ST_ = 0.086), although less so than African and South American population of *S. globulifera* (*F*_ST_ = 0.310) (Torroba‐Balmori et al., 2017), or even than Neotropical populations of *S. globulifera* (*F*_ST_ = 0.138) (Dick & Heuertz, 2008). The two ecotypes follow thus different evolutionary paths and may show genetic variants associated with adaptations to different habitats, yet genetically intermediate individuals (F1, F2, etc.) have been observed (N. Tysklind—unpublished data), suggesting that some mixing of the two ecotypes occurs in the field.

Previous shadehouse common garden experiments (Baraloto et al., 2007) pinpointed physiological differences between the two ecotypes, although none of them showed clear responses to drought and flooding compared to controlled conditions. Thus, the heterogeneous spatial distribution of *Symphonia* is not explained by these alone, at least as far as experiments in artificial conditions can tell. Nevertheless, mortality was higher in *S*. *sp1* after 6 weeks of controlled flooding, and wild *S. globulifera* seedlings tended to survive better in SF areas in the wild (Baraloto et al., 2007). To dissect the causes of the observed association of the two ecotypes with habitats, we established an RTE, considering both habitat and provenance region, in the field under the natural canopy. We hypothesize that each ecotype and provenance exhibits higher fitness, as measured by germination, growth, survival, herbivory defense, or a combination of these factors, in its habitat of origin than in other habitats; we argue that such differences contribute to explain the niche distribution patterns observed in the wild.

## 2 MATERIALS AND METHODS

### 2.1 Experimental design and data collection

**FIGURE 2:**
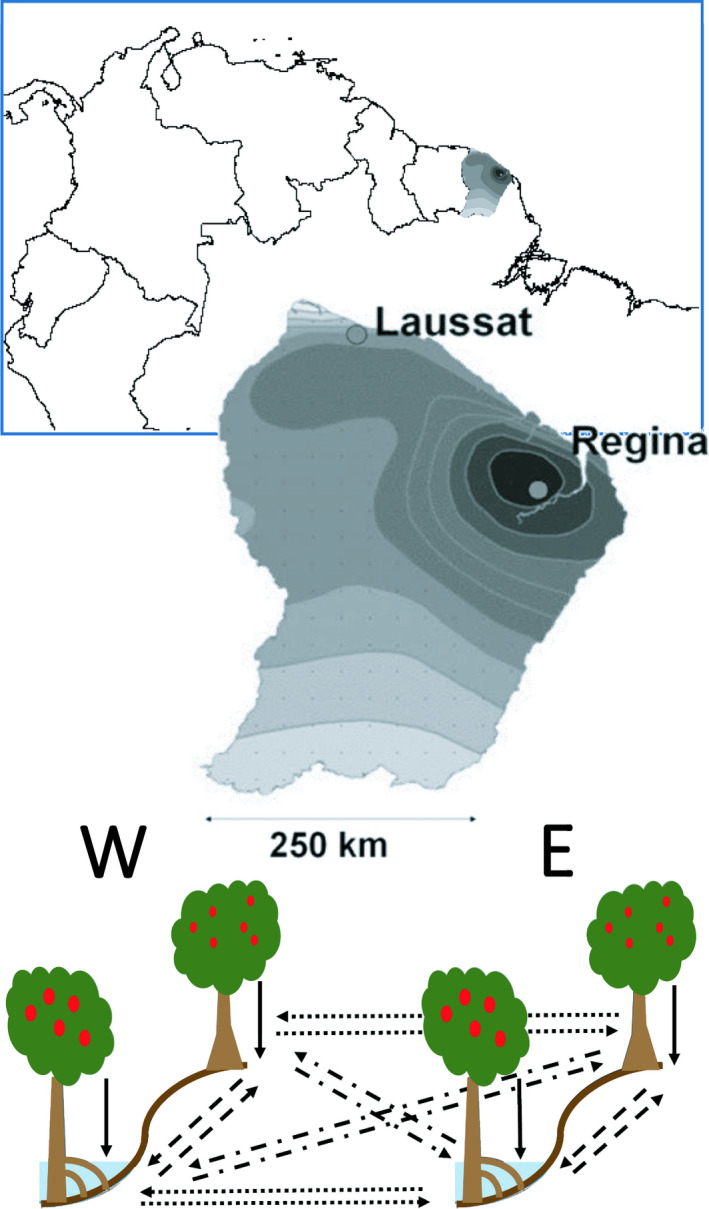
Geographical location, pluviometry, and design of reciprocal transplantation experimental sites. (a) Location of French Guiana along the north of South America; (b) grayscale map of pluviometry across French Guiana and location of the two plantation sites (West: Laussat and East: Régina). Average rainfall: Laussat (2,500 mm/year), Régina (3,500 mm/year) (reproduced from Brousseau et al., 2015 with permission). (c) Design of the reciprocal transplantation experiment. The black sigmoid curves represent the edaphic difference between HT and SF; the details on cartoon trees represent the different morphologies found in *Symphonia* associated with the HT and SF (i.e., *S. sp1* in HT and *S. globulifera* in SF); solid lines represent individuals planted in the same habitats and region as their provenance. Dashed lines represent transplants between habitat within regions. Dotted lines represent transplants between regions within habitats. Dotdashed lines represent transplants across regions and habitats

The experimental design aimed to collect seeds from mother trees from two contrasting habitats and the associated ecotypes: SF‐*S. globulifera* and HT‐*S. sp1*; and from two broad regions with markedly different rainfall patterns: “east,” with the highest rainfall in French Guiana, and “west,” the driest part of French Guiana (Figure 2). Seeds were transplanted onto experimental gardens installed on HT and SF habitats at two field sites in the “east” and “west” regions, respectively, which were not among the sampled sites for the seeds.

**FIGURE 3:**
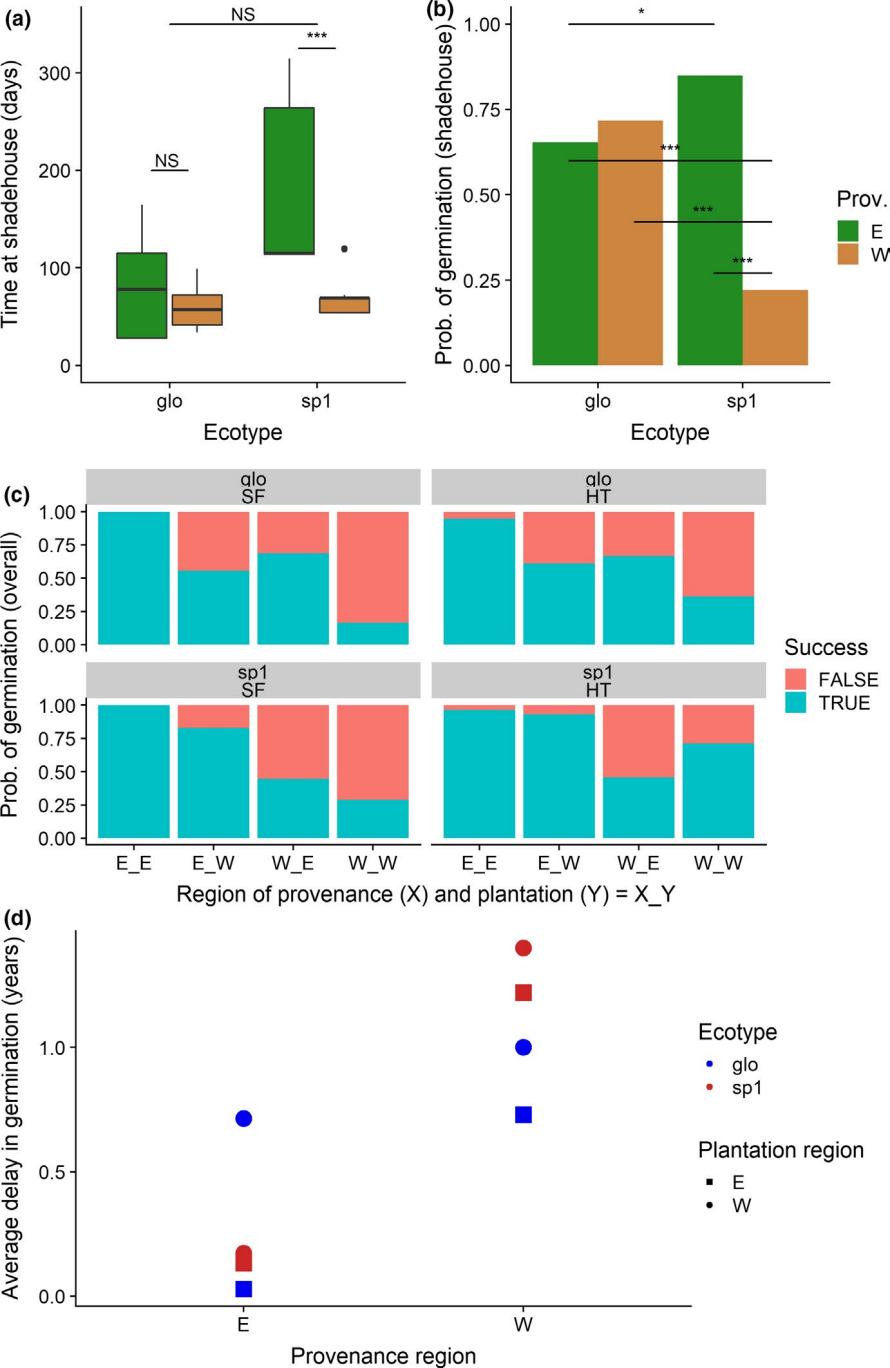
Analysis of germination success of *Symphonia* seeds. (a) Time spent in the shadehouse before transplantation to the gardens by ecotype and provenance. (b) Probability of germination in the shadehouse before transplantation by ecotype and provenance. (c) Overall probability of germination success by ecotype, provenance, habitat, and plantation. Ecotypes can refer to either *S. globulifera* (glo) or *S.sp1* (sp1); habitat can be either Hilltop (HT) or seasonally flooded (SF); region of provenance (Prov.) and plantation can be either east (E) or west (W)

Seeds were collected between September 2008 and April 2009, due to large differences in flowering times, from nine mother trees belonging to both ecotypes in the “western” region and from five mother trees in the “eastern” region, composing the variables “Provenance region” and “Ecotype” (Table 1). From each mother tree, 35–39 seeds were collected and sown in polypropylene germination plates with soil in a common shadehouse at the Kourou Agronomic Campus prior to transplantation into field sites between May and July 2009. This meant that seeds spent between 27 and 315 days in the shadehouse, depending on the seed collection and transplant dates. This introduced substantial differences among groups in the number of days spent in the shadehouse (Figure 3a), the proportion of seeds that had germinated (i.e., at least cotyledons emerged from ground), as well as in the developmental stage reached by the germinated seedlings, at transplantation time. Although such differences among groups are likely to originate from ecological differences in flowering time, they can be viewed as biases in the analyses. First of all, comparisons of germination trends among groups must be interpreted according to this bias; secondly, germination rates themselves must be included as an important cofactor, in its turn carrying information about amount of time spent in shadehouse, in the life‐history and growth‐associated traits analyses. See below for how biases in seed collection were incorporated into the analysis of germination rates and for how differences in germination status were considered in the analyses of other traits. Whether seeds had germinated at the moment of transplant or not was stored in the variable “transplant status.” The moment at which individual seeds were first recorded as seedlings (e.g., moment of transplant, year 1, year 2, year 3) was stored as “germination timing.” To avoid confusion, we hereafter refer to “seed” when discussing aspects regarding the phase prior to germination, “seedling” for those aspects regarding the phase after germination, and “individual” for those aspects regarding both seed and seedling phases.

**TABLE 1:** *Symphonia* individual information: the Mother tree from which the seed was collected, the sampling site and latitude and longitude where the mother tree was found

Mother tree	Sampling site	Latitude	Longitude	Provenance Region	Ecotype	Habitat of mother	Ni	Gt	Go	Alive Y6
W426	Kaw	4°33ʹ22.34″N	52°14ʹ20.00″W	East	*S. globulifera*	SF	36	27	31	18
W503	Régina	4°16ʹ00.84″N	52°09ʹ51.84″W				37	21	29	12
M837	Montagne Tresor	4°33ʹ38.21″N	52°13ʹ59.06″W		*S. sp1*	HT	36	25	30	14
W424	Kaw	4°33ʹ20.93″N	52°14ʹ21.12″W				38	38	38	17
W425	Kaw	4°33ʹ22.07″N	52°14ʹ22.76″W				39	31	37	15
W463	St Laurent	5°23ʹ28.70″N	53°39ʹ07.83″W	West	*S. globulifera*	SF	36	16	18	9
W466	Crique Naï	5°23ʹ47.03″N	53°42ʹ25.95″W				36	2	13	12
W497	Apatou	5°09ʹ27.57″N	54°20ʹ16.22″W				36	7	23	17
W498	Apatou	5°09ʹ27.58″N	54°20ʹ16.23″W				35	1	10	10
W465	Crique Naï	5°23ʹ47.04″N	53°42ʹ25.96″W		*S. sp1*	HT	36	0	8	7
W474	Montagne de Fer	5°21ʹ07.37″N	53°32ʹ48.57″W				36	0	18	14
W475	Montagne de Fer	5°21ʹ07.38″N	53°32ʹ48.58″W				36	4	20	16
W476	Montagne de Fer	5°21ʹ07.39″N	53°32ʹ48.59″W				36	0	16	15
W477	Montagne de Fer	5°21ʹ07.40″N	53°32ʹ48.60″W				37	12	21	14
Total							510	184	312	190
Percentages								36.1	61.2	37.3

Note

Two general provenance regions are indicated (e.g., east and west), as well as the ecotype to which the mother belonged (*S. globulifera* or *S. sp1*) and the type of habitat the mother tree was found: seasonally flooded (SF) and hilltops (HT). The number of seeds collected (Ni), the number of seeds germinated at the time of transplant (Gt), and that had germinated overall at the end of the experiment (Go), and the number of seedlings alive at year 6 (Alive Y6) are also tabulated.

Individuals (i.e., seed or seedling) were transplanted to gardens established at each field site (i.e., east vs. west) and habitat: three in SF and three in HT conditions in each site, totaling 12 gardens (see Brousseau, Fine, Dreyer, Vendramin, & Scotti, 2020, for the description of sites). Notice that, because *S. globulifera* and *S. sp1* are late‐succession, shade‐tolerant trees, the gardens were established under canopy cover. Field site plantation and habitat compose the variables “Plantation region” and “Habitat,” respectively (Table 2; Figure 2). Each garden was fenced from large herbivores with chicken wire. Prior to transplanting, all understory vegetation (i.e., up to 5 cm D.B.H.) was removed; the canopy was left undisturbed. Regeneration other than the transplanted seedlings was removed yearly by hand. Individuals were distributed over the twelve gardens as follows: Each garden was arranged in 44 ten‐seedling slots. In each garden, six slots were randomly attributed to *Symphonia* (the remaining slots were used for other experiments), and then, three individuals per mother plant were assigned to random positions within those slots in each garden. Individuals were allocated to different gardens depending on their provenance, ecotype, plantation region, and habitat without differentiating between those that had germinated in the shadehouse and transplanted as germinated seedlings or those transplanted as ungerminated seed (Table 2). Data for each individual (i.e., germination status at transplantation, germination year, survival, growth‐associated traits, and herbivory) were collected at transplant date and then yearly in September from 2009 until 2014, except for 2012 (Dryad database: https://doi.org/10.5061/dryad.stqjq2c1q; and TRY Plant Trait database: DatasetID 739). Individual survival was recorded as follows: 1 for seedlings found alive and 0 for ungerminated seeds transplanted to field sites and yet to germinate, and for seedlings previously living but found dead. Seedling height was measured in centimeters between the apical bud and the collar. Stem diameter was measured in millimeters at the collar in two orthogonal directions and estimated as the mean of the two measures. As an architectural trait, we selected “total number of leaves,” an indicator of seedling leafiness. Herbivory was determined as follows: Each leaf was assigned one of five classes of percentage of damaged area (0%–20%; 20%–40%; 40%–60%; 60%–80%; and 80%–100%), and then, seedling herbivory attack level was estimated as the average of the percentage of damaged area of all its leaves (Dryad database: https://doi.org/10.5061/dryad.stqjq2c1q; and TRY Plant Trait database: DatasetID 739).

**TABLE 2:** Reciprocal transplantation experimental garden information: The garden number, the latitude and longitude, the plantation region, habitat are indicated

Garden number	Latitude	longitude	Plantation region	Habitat	*T*_min_ (°C)	*T*_max_ (°C)	Precipitation 1981–2010 (mm)	Ni	Gt	Go	Alive Y6
Garden 1	5°28ʹ41.8980″N	53°34ʹ27.6000″W	West	HT	22.1	31.2	3,364	42	13	31	20
Garden 2	5°28ʹ49.9836″N	53°34ʹ31.7905″W						44	16	28	15
Garden 3	5°28ʹ51.2976″N	53°34ʹ28.1923″W						43	11	26	15
Garden 4	5°28ʹ39.7956″N	53°34ʹ40.4156″W		SF				42	12	17	3
Garden 5	5°28ʹ42.1068″N	53°34ʹ39.6138″W						42	9	18	11
Garden 6	5°28ʹ37.2936″N	53°34ʹ39.3984″W						44	11	20	7
Garden 7	4°18ʹ55.0908″N	52°14.089144ʹ″W	East	SF	22.4	31.6	2,528	42	22	34	22
Garden 8	4°18ʹ53.8848″N	52°14ʹ09.3016″W						41	19	23	15
Garden 9	4°18ʹ51.8364″N	52°14ʹ06.8656″W						42	19	31	26
Garden 10	4°18ʹ49.1364″N	52°14ʹ04.8827″W		HT				42	22	31	23
Garden 11	4°18ʹ49.5936″N	52°14ʹ04.4617″W						43	18	29	16
Garden 12	4°18ʹ49.7268″N	52°14ʹ03.3591″W						43	12	24	17
Total								510	184	312	190
Percentage									36.1	61.2	37.3

Note

Climatic conditions minimum (*T*_min_) and maximum temperatures (*T*_max_) and average precipitation (mm) between 1981 and 2010 at nearby stations, Regina (east) and Iracoubo (west), are indicated (Meteo France). The habitat type of each garden is also indicated: seasonally flooded (SF) or hilltop (HT). The number of seeds planted in each garden (Ni), the number of seeds germinated at the time of transplant (Gt), and that had germinated overall at the end of the experiment (Go), and the number of seedlings alive at year 6 (Alive Y6) are also tabulated.

### 2.2 Data analyses

Two complementary analytical strategies were applied to the data to extract the biological significance of individual germination, survival, and performance depending on the studied predictor variables: (a) Linear model and generalized linear model (GLM) were used to find general effects of predictor variables on the dependent variables. More specifically, we introduce a test based on the least squares means, also named adjusted means (Searle, Speed, & Milliken, 1980), which allows us to compare the effect of growing in their “home” habitat vs. “away” habitat and of being “local” vs. “foreigner” in a given habitat, while averaging for other potential effects to make such comparisons meaningful and reduce the confusion of effects. And (b) random forest methods were used to explore variable importance, untangle, and understand the structure of interactions among the covariates, and graphically visualize their effects on the dependent variables.

#### 2.2.1 Linear models and general linear model analyses

Linear model (LM) and generalized linear model (GLM) were used to test the potential effects of different predictor variables on traits (i.e., germination, survival, growth, architecture, and herbivory) and most importantly to define an ad hoc procedure to test local adaptation by subsuming in a single test both the “home vs. away,” and the “local vs. foreigner” tests of Kawecki and Ebert (2004), while averaging over all possible other effects to disentangle the effect of the unbalanced final design. Our approach, as described below, aims at synthetically observing the effect of having been planted in the environment of provenance or in a different environment, in itself, on traits taken as proxies for individual performance. This contrasts with previous strategies for the detection of local adaptation, which rest on the separate analysis of the “home vs. away” and “local vs. foreigner” effects and deduce the presence of the effect from slope comparisons (i.e., they test for population × environment interactions). In the wording of Kawecki and Ebert (2004), our method is tantamount to comparing the means of “‘sympatric’ and ‘allopatric’ deme‐habitat combinations.” While confounding the effects of “true” local adaptation and of global superiority of one population relative to all others (Kawecki & Ebert, 2004), our LM/GLM approach has the comparative advantage of better coping with unbalanced design and, possibly, having greater power.

We denote *e* the ecotype (*e* = 1 for *S. globulifera* and *e* = 2 for *S. sp1*), *h* the habitat (*h* = 1 for SF, and *h* = 2 for HT, so that individuals growing at “home” are specified by 11 or 22), *o* for provenance region (*o* = 1 for east and *o* = 2 for west), *r* for the plantation region (*r* = 1 for east and *r* = 2 for west), *s* for the transplantation status (*s* = 1 if transplanted as a seed or *s* = 2 for transplanted as a seedling), and finally, *a* for the age of the considered individual (*a* = 1, …, 5). The full model for growth‐associated traits (i.e., seedling height, stem diameter, leafiness, and herbivory) might be expressed as a normal response *Y_ehorsa_ ~ N*(*μ_ehorsa_*, *σ* ^2^), while binary responses like the life‐history traits, germination, and survival are expressed as a Bernoulli distribution with probability of success *p_ehorsa_*. In the following text, we show the development of formulas for the ecotype/habitat case, with subscripts *e* for ecotype and *h* for habitat, as described above; formulas for the provenance region/plantation region case are identical, except that they bear subscripts *o* for provenance region and *r* for plantation region, as described above, and will not be further described here.

1. Germination success in the shadehouse and overall germination (*G*)

In the evaluation of the difference in germination success (*G*) in the shadehouse, individuals have not yet been transplanted; therefore, the only effects to account for were genetic effects: ecotype and provenance region. However, the time spent in the shadehouse depends on the collection and should be accounted for. The logit of the probability of germination (*G*) of the *k*th seed is given by:

(1) $$\text{logit}\left(p_{\mathrm{eok}}^{G}\right)=\mu^{G}+\alpha_{e}^{G}+\delta_{o}^{G}+\varepsilon_{\mathrm{eo}}^{G}+\left(\beta +\gamma_{e}^{G}+\eta_{o}^{G}\right)t_{\mathrm{eok}}$$

where *t* stands for the time spent in the shadehouse.

To correct the effects of unbalanced design and difference in time in the shadehouse, the effect of ecotype (*e*) on the difference in germination success (*G*) in the shadehouse has been studied by a comparison of the classical least square means of ecotype:

(2) $$\text{logit}\left(p_{e}^{\text{adj},G}\right)=\mu^{G}+\alpha_{e}^{G}+\left(\beta^{G}+\gamma_{e}^{G}\right)\bar{t}+\frac{1}{2}\sum_{o}\delta_{o}^{G}+\xi_{\mathrm{eo}}^{G}+\eta_{o}^{G}\bar{t},$$

where ($\bar{t}$) is the average time spent in the shadehouse.

The same approach using least square means is used to study the overall germination except that we do not account for the time in the shadehouse. In case of germination success, a GLM approach is used to study the time of germination. The response is modeled through a geometric distribution and the log link function. Least square means are used to compare the expected time of germination.

1. Survival (S)

To evaluate differences in survival (*S*) among “home” and “away” groups, we developed the following approach: Assuming the survival probability is constant over a year, the observed maximal age might be modeled as a geometric distribution whose probability of success, *p^S^_ehors_*, depends on ecotype (*e)*, habitat (*h*), provenance region (*o*), plantation region (*r*), and the transplantation status (*s*). The least squares means for the log odd ratio of such probability is given by:

(3) $$\text{logit}\left(p_{\mathrm{eh}}^{\text{adj},S}\right)=\mu^{S}+\alpha_{\mathrm{eh}}^{S}+\frac{1}{8}\sum_{o,r,s}\delta_{\mathrm{ots}}^{S}+\zeta_{\mathrm{ehors}}^{S},$$

where *α^S^_eh_* stands for the joint effect of ecotype and habitat, *δ^S^_ors_* stands for all other effects like the provenance region, plantation region, and the transplantation status, and $\zeta_{\mathrm{ehors}}^{S}$ the interaction between those different effects on the survival probability.

1. Growth‐associated traits

As the aim of the analysis of growth‐associated traits is identifying the potential effect of the growing “home” versus “away,” the mean *μ_ehorsa_* has to reveal the joint effect of ecotype and habitat; the joint effect of provenance region, plantation region, and transplantation status; and the age effect and all interactions between any two of these variables. Therefore, for any growth‐associated trait (*Y*), *μ_ehorsa_* might be expressed as:

(4) $$\mu_{\mathrm{ehorsa}}^{Y}=\mu^{Y}+\alpha_{\mathrm{eh}}^{Y}+\delta_{\mathrm{ors}}^{Y}+\left(\beta^{Y}+\gamma_{\mathrm{eh}}^{Y}+\theta_{\mathrm{ors}}^{Y}\right)a$$

where $\alpha_{\mathrm{eh}}^{Y}$ stands for the joint effect of ecotype and habitat, with a total of four possible different combination of ecotype × habitat, $\delta_{\mathrm{ors}}^{Y}$ stands for all other controlled effect like the provenance region, the plantation region, and transplantation status for a total of eight possible different levels, $\beta^{Y}$ is the effect of age, $\gamma_{\mathrm{eh}}^{Y}$ is the differential effect of age according to the ecotype/habitat level, and $\theta_{\mathrm{ors}}^{Y}$ is the differential effect of age according to the provenance/plantation region/transplantation status levels.

Interaction between main effects of interest (i.e., ecotype/habitat) and other controlled effects has not been incorporated as not all combinations have been observed.

To detect signals of local adaptation in growth‐associated traits, we compared the effects of growing at the ecotype's (or provenance's) “home” habitat versus growing in the “away” habitat. Such comparison was achieved by defining least squares means at age a *μ_eh_^adj^(a)* (Lenth, 2016) to account for the unbalanced design:

(5) $$\mu_{\mathrm{eh}}^{adj,Y}\left(a\right)=\mu^{Y}+\alpha_{\mathrm{eh}}^{Y}+\left(\beta^{Y}+\gamma_{\mathrm{eh}}^{Y}\right)a+\frac{1}{8}\sum_{o,t,s}\left(\delta_{\mathrm{ors}}^{Y}+\theta_{\mathrm{ors}}^{Y}a\right)$$

The comparison between “home” and “away” habitat–ecotype pairs is performed age by age by forming the following contrast:

(6) $$C_{a}=\left(\mu_{11}^{\text{adj},Y}\left(a\right)+\mu_{22}^{\text{adj},Y}\left(a\right)\right)‐\left(\mu_{12}^{\text{adj},Y}\left(a\right)+\mu_{21}^{\text{adj},Y}\left(a\right)\right)$$

which quantifies the difference of an average individual growing at “home” (combination 11 or 22) and an average individual growing “away” (combination 12 or 21).

All GLM analyses were run in the R statistical environment (R Core Team, 2020) with the packages “car” (Fox & Weisberg, 2010)⁠, “multcomp” (Hothorn, Bretz, & Westfall, 2008)⁠, “emmeans” (Lenth, 2016, 2018)⁠,⁠ and visualized using “ggplot2” (Wickham, 2009)⁠.

#### 2.2.2 Random forest analyses

*Random forest* methods and classification trees were applied to evaluate relative variable importance in explaining germination, survival, growth, leafiness, and herbivory on individuals at the end of the experiment (year 6) and the average yearly relative growth rate (RGR), untangle interactions among the predictor variables, and graphically visualize their effects on each of the responses. *Random forest* methods (Breiman, 2001) are particularly suited for data where nonlinear relationships and complex interactions among variables are expected (Cutler et al., 2007). Classification trees visualize predictive models of responses significantly dependent on predictor variables. This is achieved by recursive binary classification of the data, where independence of the response (i.e., here germination, survival, growth, leafiness, and herbivory) and the covariates (i.e., here provenance region, ecotype, plantation region, habitat, and transplant status) is tested; then, if a significant dependence is found, the best split value for the predictor variable with the strongest effect is retained and used to divide the response in two groups. The process is then repeated with each of the groups, recursively, until no significant dependence between covariates and response can be found (Hothorn, Hornik, & Zeileis, 2006). In a *random forest,* the above classification tree is performed on a bootstrap subset of the data and a reduced number of predictor variables to obtain response predictions based on a majority vote of the whole forest. Such methodology allows assessing relative variable importance, by identifying those covariates which, when removed, ensue a significant drop of prediction power (Strobl, Boulesteix, Zeileis, & Hothorn, 2007). In our case, it allows us to identify whether certain combinations of variables (e.g., *S. globulifera* in SF) lead to significant improvement of performance of “home” or “local” individuals.

We repeated the analyses including all the predictor variables as explanatory variables and removing transplant status (*i.e.,* seed or seedling) to check whether transplant status confounded the analysis of the impact of the covariates of interest (*i.e.,* provenance region, ecotype, plantation region, and habitat). Furthermore, we evaluated the effects of provenance region and ecotype on shadehouse germination rates and of all predictor variables on the germination rates of individuals planted as seeds in the field (Appendix S1).

For the *random forest* analyses of growth performance and herbivory, only individuals germinated in 2009, whether in the shadehouse or in the field and having a final measure in 2014, were analyzed. Therefore, each individual has a unique discrete value for each trait in 2014, that is at age 5 (i.e., year 6). The average yearly relative growth rate (RGR) in height, diameter, and leafiness, as well as the average herbivory, was also summarized over the life of the individual, giving each individual a single value over the course of the experiment.

All analyses were run in the R statistical environment (R Core Team, 2020) with the packages “party” and “partykit” (Hothorn et al., 2006; Hothorn & Zeileis, 2015; Strobl, Hothorn, & Zeileis, 2009). Conditional inference trees were grown with an α = 0.05 and a minimum of 2 observations in each branch and visualized with “ggparty” (Borkovec, 2019) and “ggplot2” (Wickham, 2009).

## 3 RESULTS

### 3.1 Description of the data

A total of 510 individual *Symphonia* were followed over the course of 6 years. Of these, 36.1% had germinated at the time of transplant and were transplanted as seedlings. The remainder were transplanted as seeds. Overall germination reached 61.2% by the end of the experiment (Table 1). At the end of the experiment, 37% of seedlings were alive, and survival was lowest in western SF gardens (Table 2). Summary statistics of growth‐associated traits (i.e., height, diameter, total number of leaves) and average herbivory over the course of the 6 years for those individuals germinated in 2009 and still alive at the end of the experiment are reported in Table 3.

**TABLE 3:** Summary statistics (minimum, mean, maximum, and standard deviation (*SD*)) of growth traits and herbivory of seedlings germinated in 2009 and still alive in 2014

	Min	Mean	Max	*SD*
*H* (cm)	9.0	28.5	115.0	14.2
*D* (mm)	1.38	4.12	11.27	1.57
TNL	1	17	186	17.6
Herb_ave (%)	10	13.6	30	4.23

Note

Height (*H*), diameter (*D*), the total number of leaves (TNL), and average herbivory (yearly average of percentage of damaged area of all its leaves) over the course of the 6 years (Herb_ave).

### 3.2 Impact of the transplant status on survival: seeds vs. seedlings

The classification tree analysis of the success of germination (Appendix S1) indicated strong effects of the provenance region independently of the transplant status: seeds having germinated in the shadehouse or seeds having germinated in the field. The same result was observed using GLM when considering the global success of germination (i.e., whatever the transplant status, all the seeds that have germinated at some point, during the 6 years of the experiment). Since the main effect tested was observed in both the seeds that have germinated in shadehouse and the seeds that have germinated in the field, the two transplant statuses were merged together for the rest of the analyses.

### 3.3 Effects of covariates on germination and survival of *Symphonia* seedlings

**FIGURE 4:**
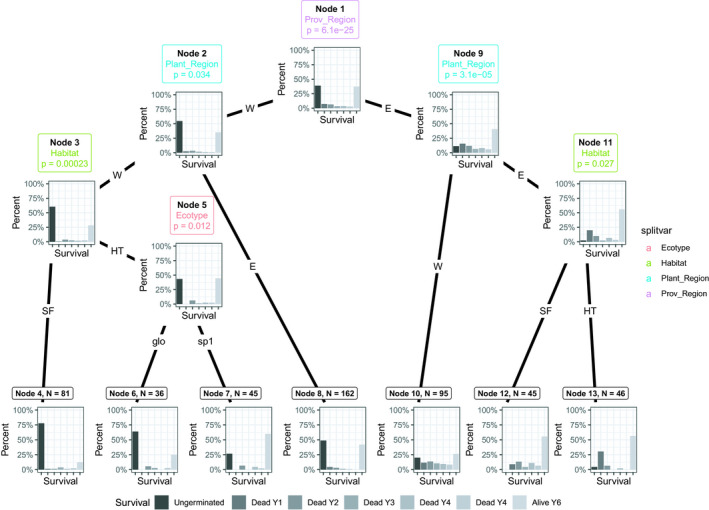
Classification tree of germination and survival of *Symphonia* seeds and juveniles after 6 years according to the covariates of interest: Prov_Region = provenance region (E = East; W = West), Plant_Region = planting region (E = East; W = West), Habitat = planting habitat (SF = Seasonally flooded; HT = hilltops), Ecotype = ecotype of the mother tree (glo = *S. globulifera*; sp1 = *S.sp1*). Individual responses (germination, survival, and mortality) were categorized as: U, ungerminated at the end of the experiment, D1‐D5, dead at year 1 to year 5, respectively, A, alive at the end of the experiment (Year 6)

To overcome the bias in duration of time at the shadehouse among the different ecotype × provenance groups (Figure 3a), we compared the probability of germination success in the shadehouse of the two ecotypes depending on the region of provenance after the average time in the shadehouse (i.e., 89 days). A significantly lower probability of germination success in the shadehouse was identified for western *S. sp1* compared to all other groups (Figure 3b), even when accounting for the difference in time in the shadehouse. However, the analysis of overall germination (i.e., shadehouse and field combined) indicated that provenance and plantation habitat, rather than ecotype, were the main drivers of the variance in germination success (Figures 3c and 4). The analysis of the delay on germination showcases that provenance also had a strong impact on the timing of germination (Figure 3d), where seeds from the west germinate significantly later than those from the east (west: 13 months vs. east: 3 months).

In terms of overall germination and survival, classification trees identified provenance region, plantation region, habitat, and ecotype as significant variables explaining overall germination and survival of individuals at the 5% threshold, exposing the complexity of the interactions among predictor variables (Figure 4). Provenance had the strongest impact, where most individuals from the east (Figure 4, node 9 bar plot) had germinated, while those from the west systematically suffered from lower germination rates (Figure 4, node 2 bar plot). Eastern provenance individuals planted in the east plantations survived significantly better than when planted in the west (Figure 4, node 9, compare bar plots at nodes 10 and 11). There was a significant effect of habitat in eastern individuals planted in the east, where 100% of seed germinated in the SF, and differences in the yearly mortality rates (Figure 4, node 11, compare bar plots at nodes 12 and 13), although both groups had similar survival rates after 6 years (~55%). Western provenance individuals survived better in the east than in the west (Figure 4, node 2; compare bar plots at nodes 3 and 8). Germination and survival were lower, for western provenance individuals, planted in the west in SF habitats than in HT habitats, regardless of ecotype (Figure 4, node 3; compare bar plots at nodes 4 and 5). Finally, for western provenance individuals, planted in the west, in HT habitats, there is a significant ecotype effect, where *S. sp1* (i.e., “local,” as deduced from adult tree distributions in natural forests, see Introduction; Allié et al., 2015; Schmitt et al., 2020)) germinates and survives significantly better than *S. globulifera* (i.e., “foreign”) (Figure 4, node 5; compare bar plots at nodes 6 and 7).

### 3.4 Effects of predictor variables on growth‐associated traits of *Symphonia* seedlings

**FIGURE 5:**
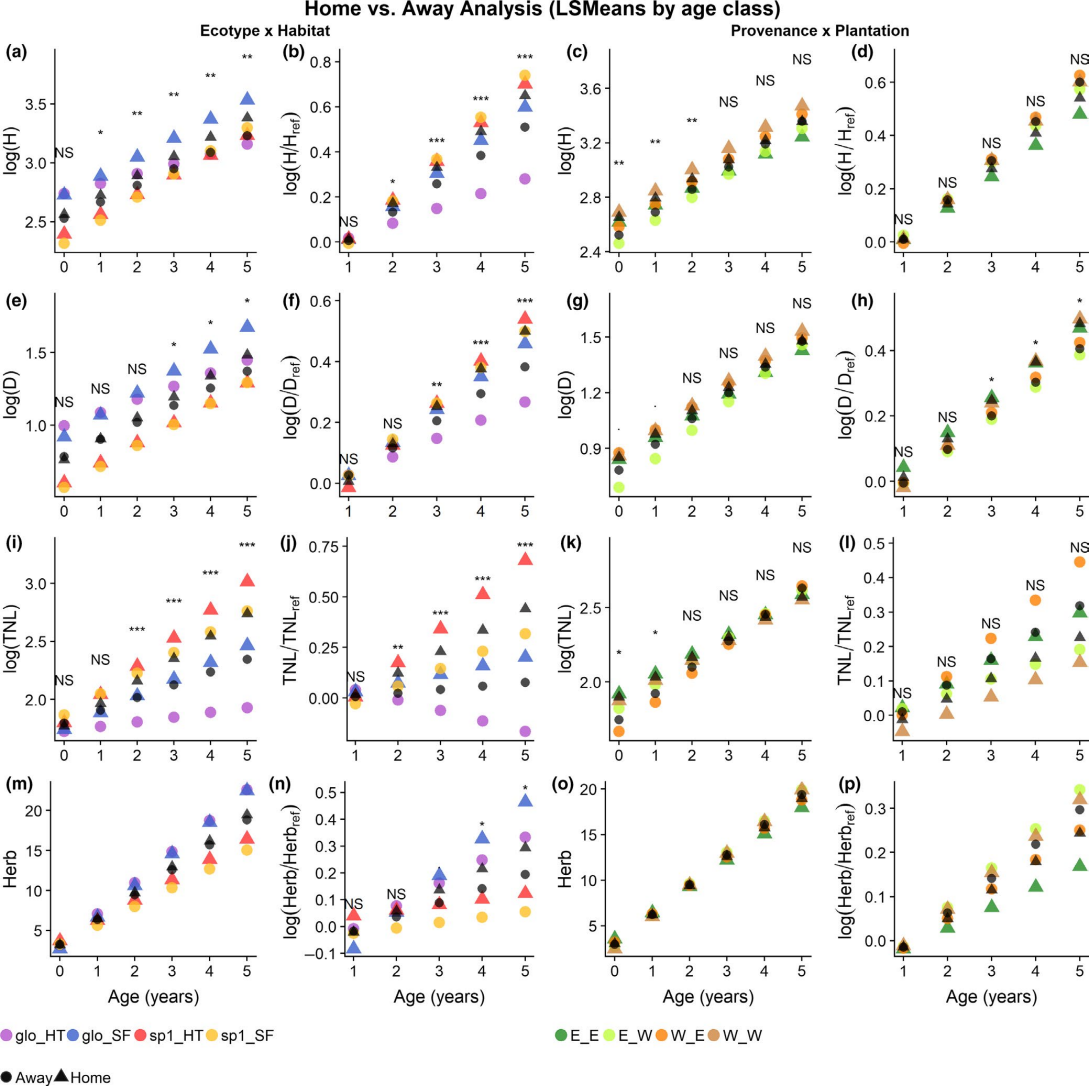
Least square means yearly analysis of growth traits (height (*H*), diameter (*D*), total number of leaves (TNL)), and herbivory (Herb) of *Symphonia* seedlings. Age in years in on the *x*‐axes. Log of growth traits and herbivory, and log of growth traits and herbivory at age compared to that at age 0 are on the *y*‐axes. Two comparisons are shown: Ecotype × Habitat (a,b,e,f,i,j,m,n) and Provenance × Plantation (c,d,g,h,k,l,o,p). Ecotypes: *S. globulifera* (glo) and *S. sp1* (sp1). Habitats: hilltops (HT) and seasonally flooded (SF). Provenance: east (E) and west (W). Plantation: east (E) and west (W). Groups growing in their “home” environment relative to the comparison are denoted by a triangle. Groups growing in “away” environments relative to the comparison are denoted by circles. Significance: nonsignificant (NS), 0.05 (*), 0.01 (**), 0.001 (***)

The linear model analyses, where LS Means were used to correct for unbalanced design, of the yearly comparison of growth‐associated traits contrasting “home” versus “away” (i.e., ecotype × habitat and provenance × plantation combinations) revealed significant interaction effects on the performance of seedlings. Figure 5 illustrates the significantly better growth performances (e.g., height, diameter, and TNL, as well as their relative growth compared to reference measures at age 0 (X/Xrel)) of seedlings grown in their “home” ecotype × habitat combination compared to those in “away” ecotype × habitats combination and that the differences increase with age (Figure 5a,b,e,f,i,j).

The provenance × plantation analyses showed significant effects in the early ages for height, diameter (marginally significant), and total number of leaves, where “home” individuals outperformed “away” individuals, but the significance disappeared in later years (Figure 5c,g,k). Individuals grown in their “home” provenance × plantation combination had significantly larger diameters at ages 3–5 relative to their diameter at age 0 compared to individuals in “away” provenances × plantation combination (Figure 5h).

**FIGURE 6:**
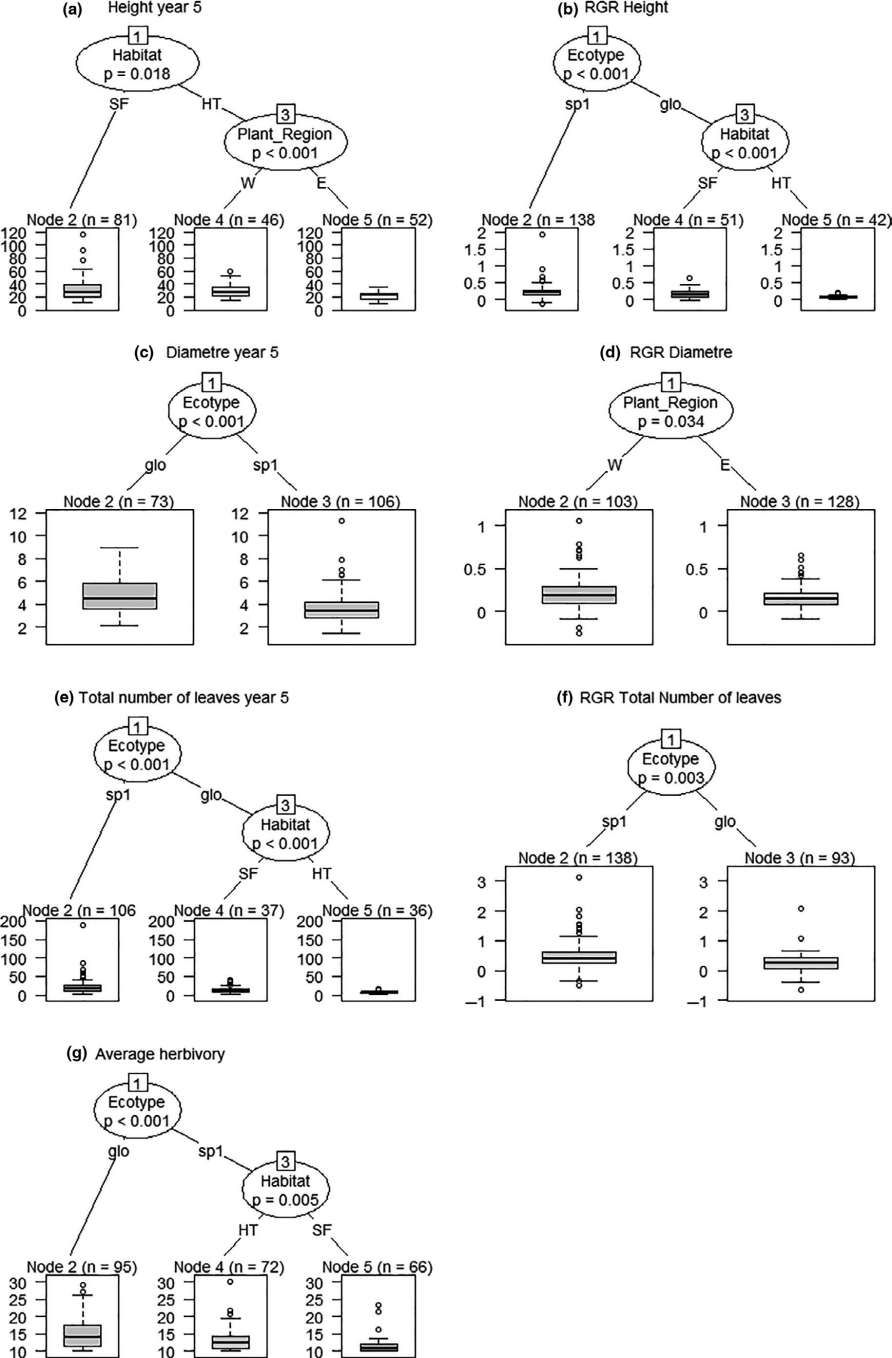
Classification tree analyses of juvenile phenotypic traits according to the studied covariates of interest. Prov_Region = provenance region (E = East; W = West), Plant_Region = planting region (E = East; W = West), Habitat = planting habitat (SF = Seasonally flooded; HT = hilltops), Ecotype = ecotype of the mother tree (glo = *S. globulifera*; sp1 = *S.sp1*). Measured phenotypic traits: H = height at age 5, RGR_H = relative growth rate in H; D = diameter at age 5, RGR_D = relative growth rate in D; TNL = total number of leaves, RGR_TNL = relative growth rate in TNL; HERB_AVE = average herbivory over the course of the experiment. Only individuals germinated in 2009 (shadehouse or field) were included. Only individuals alive in 2014 were included for measures at age 5. Only individuals with at least two annual measures were included for RGR analyses

According to the classification trees, ecotype had the strongest significant effect on 5 out of 7 growth‐associated traits and herbivory: average RGR height, diameter, total number of leaves, and average RGR in number of leaves, and average relative herbivory, separating *S. globulifera* from *S. sp1* (Figure 6). *S. sp1* grew faster (Figures 5a,b and 6b), was thinner (Figures 5e,f and 6c) but leafier (Figures 5i,j and 6e), and suffered less herbivory than *S. globulifera* (Figures 5n and 6g). Habitat also had significant effects on four growth‐associated traits, revealing that: Individuals were taller on average in SF than in HT (Figure 6a), that *S. globulifera* grew faster and was leafier in SF than in HT (Figure 6b node 3, 6e node 3), and that *S. sp1* suffered the least herbivory when planted in SF habitats (Figure 6g, node 3, Figure 5n). Finally, plantation region had an impact on the height of individuals planted in HT (Figure 6a, node 3) and on the RGR in diameter (Figure 6d), with individuals planted in the west being taller and thicker than those in the east.

## 4 DISCUSSION

In a way, our two *Symphonia* ecotypes behave in a species‐like way relative to habitat preferences, given that they can grow in mixed or neighboring stands, where morphological and genetic hybrids are occasionally found, yet they retain their respective ecological properties. Superior performances of “home” and “local” individuals in life‐history and growth‐associated traits suggest that *Symphonia* trees have locally adapted to different environmental conditions across French Guiana. We find both “home” versus “away,” and “local” versus “foreigner” examples of local adaptation (sensu Kawecki & Ebert, 2004). The patterns are, however, complex, revealing that the measured traits are not exclusively caused by genetic trade‐offs in the underlying genes coding for the patterns of local adaptation.

### 4.1 Patterns of germination

No clear environmentally cued dormancy mechanism has been so far identified in *Symphonia*. The classification trees and the estimated shadehouse and overall probability of germination (Figure 3 and Figures S3–S5) exposed some unexpected patterns: Western *S. sp1* seeds have a significantly lower probability of germination under the controlled conditions of the shadehouse after 89 days than all other groups (Figure 3b). However, overall germination at the end of the experiment of western *S. sp1* planted in HT in the west was relatively high (~70%, Figure 3c), indicating that germination for western *S. sp1* in western HT recovered while in the field. The germination of western *S. sp1* in other habitat–region combinations remained very low till the end of the experiment (Figure 3c). In stark contrast, all four eastern provenance combinations of ecotype and habitat planted in the east had nearly 100% overall germination success. Western seeds germinated significantly later than eastern seeds (Figure 3d: 1 year later on average). Such differential success rate and timing of germination among ecotype–provenance combinations could be due to differences in local adaptation to germination timing and cues. Matching germination with the best possible conditions for seedling growth is paramount for seedling survival; however, the timing and environmental cues underpinning such favorable conditions may vary across a species range. Variation in seed dormancy duration, and the genetic basis for such variation as opposed to just phenotypic plasticity, has been reported as evidence for local adaptation among populations of *Arabidopsis thaliana* (Donohue, 2009; Postma, Lundemo, & Ågren, 2015). Similarly, the regional differences in germination success over the course of our study may indicate evolutionary advantages for delayed germination of *Symphonia* in the west or rapid germination of *Symphonia* in the east (Figure 3d). A higher seed quiescence or dormancy level, or tighter environmental requirements for germination, may have emerged as a local adaptation in western *Symphonia*, especially in *S. sp1¸* as a means to cope with a drier environment (Dalling, Davis, Schutte, & Elizabeth Arnold, 2011), and spreading seedling mortality risk across several years (Gremer & Venable, 2014). Supporting such hypothesis, we observe reduced survival of eastern provenance individuals in the west (Figure 4, nodes 10 vs. 11) indicating that the western plantations are in a harsher environment overall. Conversely, a quicker germination time may be a local adaptation in response to differences in seed mortality rate (e.g., herbivory, disease, or aging) between regions or ecotypes (Dalling et al., 2011; Postma et al., 2015).

### 4.2 Patterns of growth‐associated traits

The LS Means analyses exposed how individuals in their home habitat significantly outperformed individuals in away habitats in terms of growth‐associated traits, and the classification trees pinpointed how that signal was dominated by significant decreases in growth‐associated traits for *S. globulifera* when planted in HT, indicating a reduction in competitive growth performance of individual *S. globulifera* in HT. Conversely, we did not detect any significant effect of habitat for *S. sp1*, suggesting a capacity to perform well regardless of habitat. Individual tree vigor, as in the difference between observed and expected growth, has been shown to have a pervasive effect on Neotropical tree survival, where variance in individual vigor along the tree's life was the most important variable predicting survival, well above ontogenetic status or species membership (Aubry‐Kientz, Rossi, Boreux, & Hérault, 2015). Variance in growth performance, such as seen in *S. globulifera,* can be interpreted as variance in vigor, which could be one of the mechanisms explaining the variance in survival patterns we observed among *Symphonia* seedlings. The observed variance in growth is indeed in accordance with the rarity of adult *S. globulifera* in HT habitats and the more generalist distribution of *S. sp1* across habitats (Allié et al., 2015; Schmitt et al., 2020).

### 4.3 Limitations of the methods

The power of our tests and the meaning of their results may suffer from multiple biases. Differences in germination successes lead to unbalances. While the analytical method we developed is meant to compensate them, they may still affect the results. Maternal effects (e.g., maternal provision to seeds) may still influence seedling growth and resources, because seedling mass is probably still in the same order of magnitude as seed mass. We sampled a relatively small number of maternal families from each ecotype, which may lead to biases in the assessment of genetically based phenotypic diversity within each ecotype. Nevertheless, the fact that phenotypic ecotype differences are stable in common shadehouse experiments (Baraloto et al., 2007), and in field experiments (our data, Figure 1), suggests that differences between ecotypes are consistently larger than variations within ecotype. This should make extensive sampling of within‐ecotype diversity less critical, as far as the study of ecotype differences is concerned. In addition, to make sure that ecotypes differ more than families within ecotypes in our data, we have carried out an analysis (Appendix S2) on morphological traits, confirming the patterns found by Baraloto et al. (2007). Epigenetic inheritance may also contribute to differences in seedling reactions to environmental cues. In the absence of precise information about genetic divergence and gene expression/regulation differences between ecotypes, it is hard to tell which mechanism is at play in the *Symphonia* system. Finally, as stressed by Miglia et al. (2005), to gain a comprehensive understanding of the ecological factors driving survival and performance of related taxa across environmental variables, multilife‐stage comparisons including germination, somatic growth, and reproduction should be included.

### 4.4 Patterns of survival and contributions toward understanding the patterns of species distribution

The classification trees of combined germination and survival revealed complex interactions among the predictor variables in determining the patterns of survival of *Symphonia* individuals in our experiment. The direction of the detected effects is compatible with reasonable expectations on how ecological filtering of phenotypes may operate in natural forest dynamics, pointing at mechanisms contributing to patterns of ecotype distribution in French Guiana and to the maintenance of ecological differences between ecotypes within *Symphonia*.

The groups with the highest survival (>50% after 6 years) were western provenance *S. sp1* planted in western HT (Figure 4, node 5) and eastern provenance individuals planted in the east regardless of ecotype and habitat (Figure 4, node 11). These two high survival groups also had high germination rates (Figure 3c). The first group is suggestive of very specific local adaptation. It is the only group planted in the west with a relatively high survival (i.e., >50% survival at age 5, compared to <25% for the rest of groups transplanted in the west). *S. sp1* is common in hilltops throughout French Guiana and may therefore be able to cope better in the drier west. Furthermore, it is only western *S. sp1* which significantly separate from all others in terms of survival, perhaps indicative not only of an ecotype adaptation to drier HT, but also a regional effect where eastern *S. sp1* are particularly drought tolerant. This case constitutes a double example of a “local vs. foreign” evidence of local adaptation across two variables (both habitat and regional), stressing the efficacy of the selection pressures in eastern HT habitats. Variations in survival and germination in *S. sp1* are furthermore accompanied by an overall lower performance of *S. globulifera* seedlings when planted in HT, both probably contributing to contrasted distributions between the two ecotypes. We did not detect an adaptive cost for *S. sp1* in the form of lower survival in either eastern gardens nor in SF habitats, suggesting that either (a) conditional neutrality (i.e., whereby an adaptation conveys a performance advantage in one environment without costs in alternative environments) is at play in the genetic basis underlying its improved performance in dryer conditions (Anderson, 2013; Wadgymar et al., 2017) or that (b) our experimental design did not capture the selective pressures penalizing *S. sp1* in wetter conditions, such as those found in SF habitats or eastern gardens. The latter could be related to environmental variables we did not account for in the experiment or related to stages we missed across the trees’ life history (Miglia et al., 2005).

The second group with comparatively high survival confirms a better performance of eastern individuals in eastern field sites compared to western field sites, indicative of either local adaptation at the regional level to heavier rainfalls or, alternatively, poor drought tolerance, as the survival of eastern provenance individuals, regardless of ecotype, planted in the west drops significantly. This constitutes an example of “home vs. away” pattern of local adaptation, but not “local vs. foreign,” as western provenance individuals have a high survival in the east once germinated. Given the general high survival of individuals in east plantation gardens and the nonappearance of other factors significantly affecting survival in these gardens, we infer a less stressful environment in the eastern field site for *Symphonia* in general. We did not capture evidence of differences in survival between ecotypes or habitats in the east, which exemplifies the potential confusion between divergent selection and differences in habitat quality.

Overall, we find evidence that *S. sp1* has better survival in the driest conditions, suffers less herbivory, and has no penalization on other environments, which suggests a habitat generalist behavior and matches the extant species distribution (Allié et al., 2015; Schmitt et al., 2020). Conversely, *S. globulifera* is triple penalized out of SF (i.e., lower TNL, lower RGR in height, and higher herbivory), suggesting that it is a habitat specialist limited to SF habitats. The variance in juvenile performance in the two habitats may contribute to the maintenance of the ecological differences between ecotypes.

### 4.5 Selective pressures behind the signals of local adaptation

The experimental setup was designed to detect adaptation patterns to the combined effects of the contrasted habitats, with a focus on soil factors influencing soil water availability. Our results show a pattern consistent with an adaptive advantage of western *S. sp1* to the driest conditions included in the experiment (i.e., western HT), which may be caused by differences in rooting system structure, water use efficiency (Baltzer, Thomas, Nilus, & Burslem, 2005), or a variety of other traits related to response to drought.

Beyond the sharp variations in soil water availability, many other variables covary across the microhabitats presented here (i.e., east vs. west, SF vs. HT): access to resources such as light and soil nutrients, the floristic and soil microbiota community, and presence of herbivores vary significantly between HT and SF.

Trees living in SF habitats double their risk of death through tree fall (Ferry, Morneau, Bontemps, Blanc, & Freycon, 2010), but experience a higher access to light, due to gaps created by tree falls, and a higher soil fertility, creating a high‐risk high‐gain environment. Species specializing in SF habitats must therefore adapt their resource allocations accordingly. *S. globulifera* seedlings in SF were the tallest (Figure 5a) and had the largest diameters after 6 years (Figure 5e), potentially indicative of an ecotype adaptation toward a strategy maximizing growth in a risky environment. Supporting this hypothesis, Schmitt, Hérault, et al. (2020) found that adult *S. globulifera* have leaf functional traits typical of an acquisitive strategy, contrasting those of *S. sp1*, which has leaf functional traits typical of a conservative strategy.

Arthropod assemblages in French Guiana SF and HT habitats are significantly different, where leaf feeders in particular are more abundant in HT than in SF (Lamarre et al., 2016). Herbivory was highest for *S. globulifera* regardless of all other covariates and lowest for *S. sp1* in SF habitats, suggesting different predator avoidance strategies between ecotypes. Our herbivory analyses are in agreement with those of previous studies, where similar patterns are observed in RTEs between species specializing in high‐ and low‐herbivory pressure environments: species which are normally exposed to a higher herbivory environment (i.e., similar to *S. sp1*), experienced reduced herbivory in low‐herbivory environments (i.e., similar to SF) compared to their “home” environment and species from low‐herbivory environments (Jennifer L Baltzer & Davies, 2012; Fine et al., 2006; Fine, Mesones, & Coley, 2004). *S. globulifera* tissues are rich in secondary metabolites of the *b*is‐xanthone family, known to have insecticidal properties in other organisms (Ondeyka et al., 2006; Wezeman, Bräse, & Masters, 2015); the leaves are particularly rich in globulixanthone E (Cottet et al., 2014), which has strong antimicrobial activity (Nkengfack, Mkounga, Meyer, Fomum, & Bodo, 2002). *S. globulifera* populations from Cameroon and French Guiana differ for their content in antimicrobial and antiparasitic compounds (Cottet et al., 2017), suggesting that chemical differences may also occur between *S. globulifera* and *S. sp1*. Under this hypothesis, *S. sp1* may have adapted to a higher herbivory environment (HT) by increasing the production of unpalatable and toxic compounds to compensate for a potential limitation in resources in HT habitats (Bryant, Chapin, & Coley, 1985; Fine et al., 2006; Fine et al., 2004). Such scenario would also explain the lower herbivory rate of *S. sp1* in SF habitats compared to HT habitats.

## 5 CONCLUSION

Our RTE experiment has given us insights into the ecological mechanisms governing differential germination and survival of cohorts of individuals in their own and foreign natural environments. We have revealed significant life‐history and growth‐associated trait differences between ecotypes and between provenances, that match with known environmental constraints (i.e., soil water and nutrient availability, death risk, and herbivory risks), and may be the result of coevolution of germination phenology and seedling survival. *S. globulifera* seedlings were penalized in HT habitats with reduced growth and higher herbivory; however, in SF habitats they outgrew other such groups (ecotypes × habitat), a pattern also observed in adults *Symphonia*, suggesting that *S. globulifera* has a specialized competitive advantage in SF habitats. Our results therefore suggest a link between differential growth and survival in seedlings and adult tree distribution and indicate that processes occurring at early life stages, far from being of an exclusively stochastic nature, contribute in a significant way to the selective processes and ecological filters that determine a species’ pattern of distribution across habitats. Furthermore, our results suggest that even relatively small environmental differences, such as those between HT and SF, can lead to the evolutionary differentiation and maintenance of distinct taxa in sympatry with different life‐history traits to suit such mosaic environmental heterogeneity despite occasional geneflow. Overall, the *Symphonia* model furthers our comprehension of the eco‐evolutionary processes underpinning the diversity and the spatial structuring of Neotropical tree communities and furthering our understating of the processes involved in the creation and maintenance of closely related taxa in sympatry.

## CONFLICTS OF INTEREST

The authors declare no conflicts of interest.

## AUTHOR CONTRIBUTION

**Niklas Tysklind:** Conceptualization (supporting); Data curation (lead); Formal analysis (lead); Supervision (supporting); Validation (supporting); Visualization (lead); Writing‐original draft (lead); Writing‐review & editing (lead). **Marie‐Pierre Etienne:** Data curation (supporting); Formal analysis (lead); Visualization (supporting); Writing‐original draft (equal); Writing‐review & editing (equal). **Caroline Scotti‐Saintagne:** Conceptualization (lead); Data curation (lead); Funding acquisition (lead); Investigation (lead); Methodology (equal); Project administration (equal); Supervision (lead); Writing‐original draft (equal); Writing‐review & editing (equal). **Alexandra Tinaut:** Formal analysis (supporting); Investigation (lead); Methodology (lead); Resources (lead); Writing‐original draft (supporting); Writing‐review & editing (supporting). **Valerie Troispoux:** Investigation (equal); Methodology (equal); Project administration (lead); Resources (equal); Supervision (supporting); Validation (supporting); Writing‐original draft (supporting); Writing‐review & editing (supporting). **Saint‐Omer Cazal:** Investigation (equal); Methodology (equal); Resources (equal); Supervision (supporting). **Louise Brousseau:** Formal analysis (supporting); Investigation (equal); Methodology (equal); Resources (equal); Writing‐original draft (supporting); Writing‐review & editing (supporting). **Bruno Ferry:** Conceptualization (supporting); Funding acquisition (supporting); Investigation (supporting); Methodology (supporting); Writing‐original draft (supporting); Writing‐review & editing (supporting). **Ivan Scotti:** Conceptualization (lead); Data curation (lead); Formal analysis (equal); Funding acquisition (lead); Investigation (lead); Methodology (supporting); Project administration (lead); Resources (supporting); Supervision (lead); Validation (lead); Visualization (supporting); Writing‐original draft (equal); Writing‐review & editing (equal).

## Supporting information

Supplementary Material

## Data Availability

All data on survival and growth of the *Symphonia* seedlings are available in the Dryad (https://doi.org/10.5061/dryad.stqjq2c1q) and TRY databases.
