# Supplementary material for: Microgeographic local adaptation and ecotype distributions: The role of selective processes on early life‐history traits in sympatric, ecologically divergent Symphonia populations
Source: Ecol Evol. 2020 Sep 17;10(19):10735–53. doi: 10.1002/ece3.6731 (PMC7548183; doi:10.1002/ece3.6731)

#### **Appendix:**

#### **Appendix 1: Impact of the transplant status on survival: seeds vs. seedlings.**

An exploratory classification tree analysis of the fate of individuals transplanted as seeds or seedlings, indicated significant differences in germination and survival between the two groups, potentially suggesting that there was a strong correlation of the transplanting status with long term survival (Supplementary Fig 1). However, the analysis of the distribution of plants transplanted as seed and as seedlings among conditions (Supplementary Fig 2), indicated that there were significant biases in the proportions of seed and seedlings in each of the condition combinations. The classification tree analysis of the success of germination (Supplementary Fig 3) and whether seeds germinated in the shadehouse or the field (Supplementary Fig 4), indicated strong effects of provenance region and ecotype, where eastern individuals had a high germination rate in the shadehouse and western individuals had low germination in the shadehouse, average field germination, and high proportion of ungerminated seeds at the end of the experiment. The analysis of germination inside the shadehouse, which removes the potential variance in germination and survival introduced by interaction with transplant environments, still shows a strong effect of the provenance region and ecotype on the germination success (Supplementary Fig 5). The analysis of the success of germination in the field (Supplementary Fig 6), also confirmed the strong effect of provenance region on germination success, as well as habitat and plantation region. The GLM analysis of the success of germination inside the shadehouse also identified a strong effect of the provenance and plantation regions (Fig. 3b), and an effect of the ecotype/habitat only through the interaction. The least square means, adjusted to correct for the unbalanced experimental design, allows the comparison of ecotype/habitat averaging over all environmental conditions. The overall germination success, in the shadehouse or in the wild, reveals an effect of ecotype/habitat and an effect of the regions of provenance/plantation using type ‘II’ test of deviance decomposition. The comparison of ecotype and habitat, when averaging over all provenance and plantation regions, using adjusted means does not show any significant differences (with p-values close to 1), which proves that the confusion between provenance and transplantation status and the effect of main interest are very high.

If we expect individuals to behave differently depending on its conditions (provenance region, ecotype, plantation region, and habitat), combined with the biased distribution of conditions among transplantation status (i.e. seeds vs. seedlings), then, analysing seeds and seedlings independently could lead to the false impression of significance of the status of transplantation in their long-term performance. If we divide data between seeds and seedlings, then, we would be accepting that the effect of growth in a shadehouse and the stress of transplantation would be stronger than the effects of provenance region, ecotype, habitat, and transplantation region, which are expected to have lifelong impacts. Finally, if we suspected a difference in behaviour between individuals transplanted as seeds vs. seedlings, we would expect a sharp increase of mortality in the first year of seedlings, which is only seen in one group (i.e. seedlings transplanted in HT in the east plantation region). Therefore, for the *random forest* and glm analyses of germination, survival, growth traits, and herbivory we decided that not including transplant status was more representative of the factors and interactions impacting individual performance in the long term.

**Supplementary Figures: Analysis of Germination:**

Supp. Fig 1: Analysis of the fate of *Symphonia* individuals according to transplant status (*i.e.* seed or seedling) and covariates of interest (*i.e.* ecotype, provenance region, planting region, and planting habitat)

**
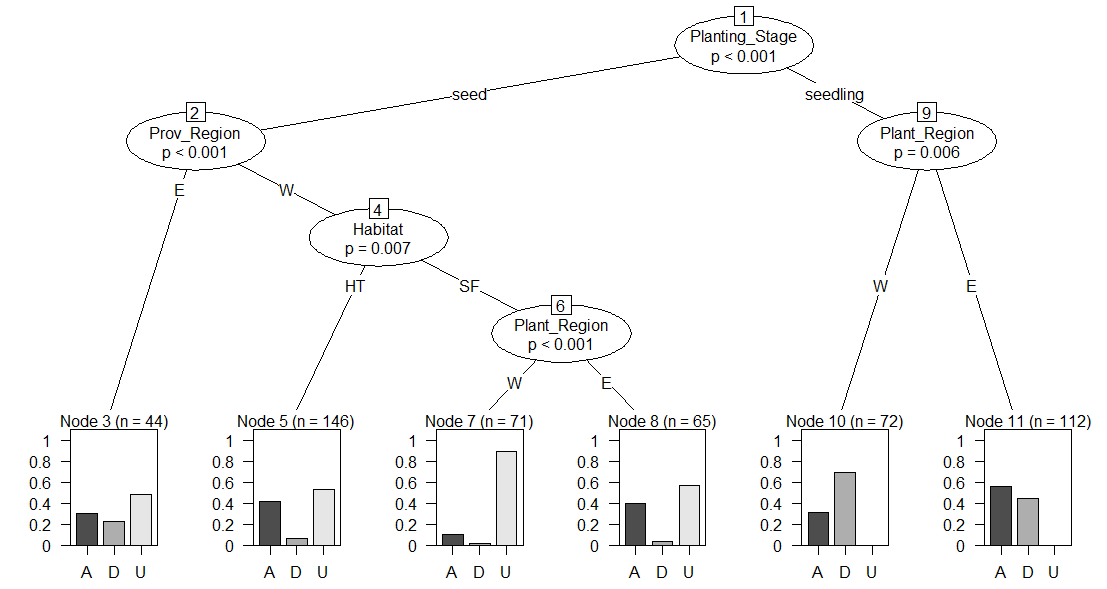
**

Supp. Fig 2: Classification tree of the distribution of *Symphonia* individuals planted as seed or seedlings according to covariates of interest (i.e. ecotype, provenance region, planting region, and planting habitat)


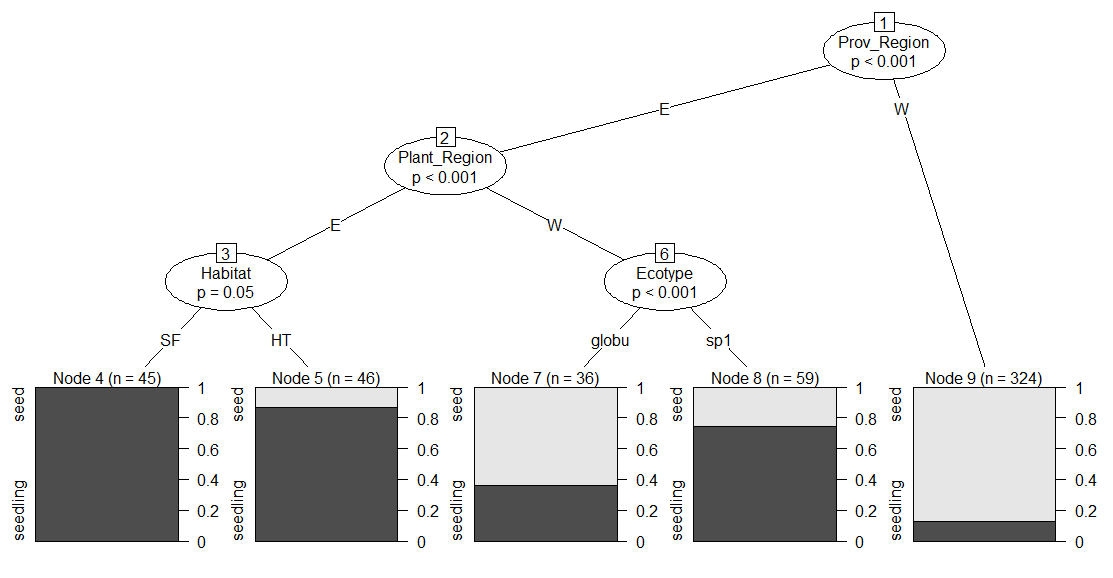


Supp. Fig 3: Analysis of overall germination success (yes or no) according to covariates of interest (i.e. ecotype, provenance region, planting region, and planting habitat)


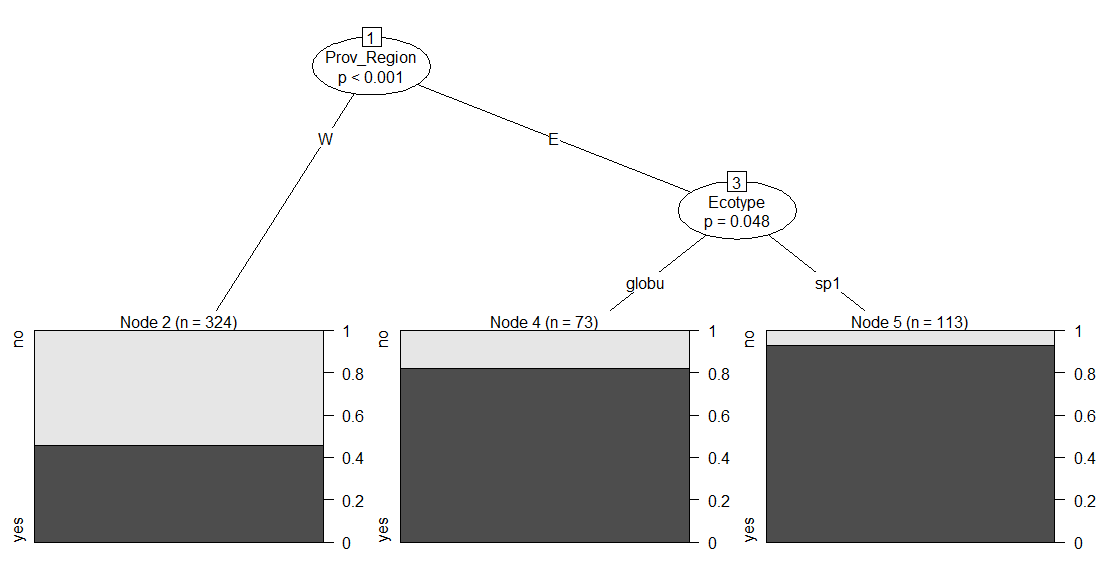


Supp. Fig 4: Analysis of germination position and success (i.e. shadehouse, field, dormant/dead) according to covariates of interest (i.e. ecotype, provenance region, planting region, and planting habitat).

**
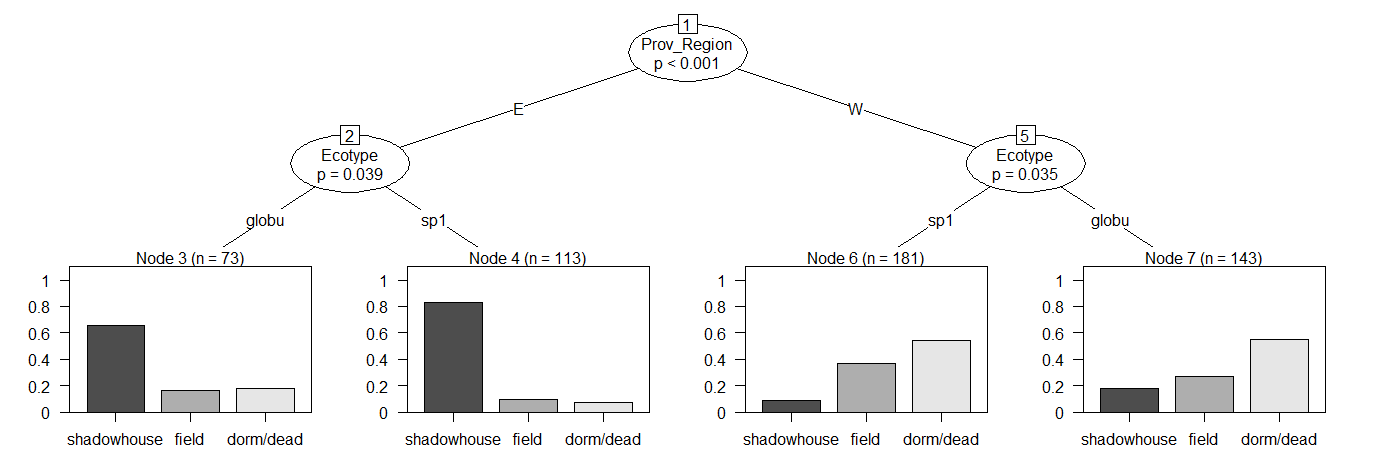
**

Supp. Fig 5: Analysis of germination success (yes or no) in the shadehouse according to covariates of interest (i.e. ecotype and provenance region).


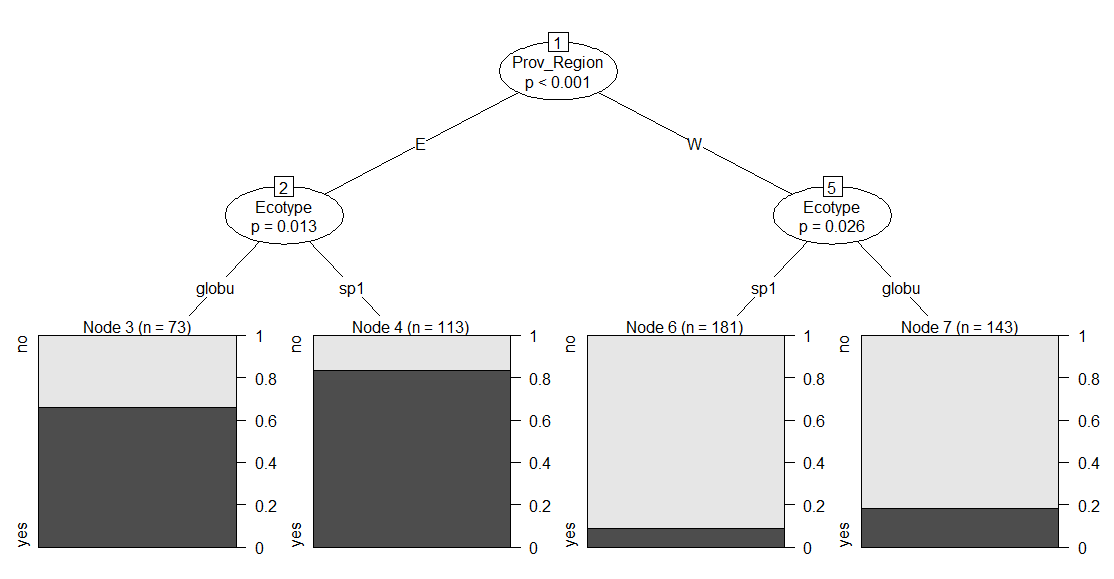


Supp. Fig 6: Analysis of germination success (yes or no) of individuals planted as seeds in the field according to covariates of interest (i.e. ecotype, provenance region, planting region, and planting habitat).


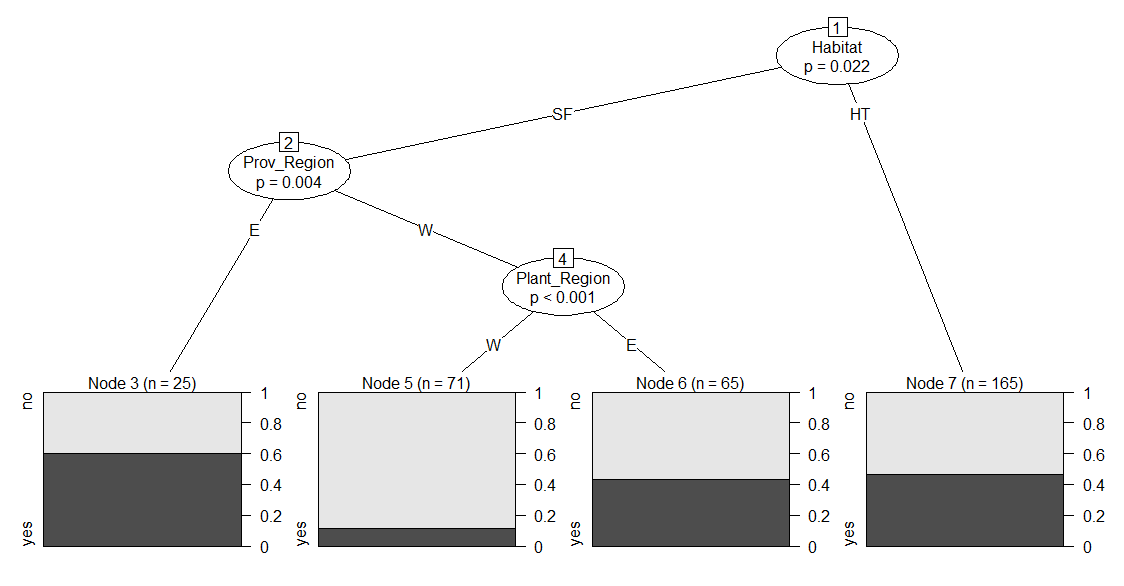


**Appendix 2 : Inspection of between-ecotype and within-ecotype allometric trait variation.**

To inspect the amount of phenotypic diversity between ecotypes, and among families within ecotypes, we carried out a simple hierarchical ANOVA with the following model:

pi = μ + ecotypei + familyi|ecotypei + εi

and then inspected the ecotype and family effects for each trait.

The analyses were carried out with R (R Development Core Team, 2008), with contrast option set to “contr.sum”.

We inspected two allometric traits known to differ between the two ecotypes: number of leafs per unit stem length and diameter/height ratio.

*S. globulifera* is expected to produce fewer, larger leaves, with shorter distances between successive leaves on the stem, resulting in a smaller number of leaves per unit stem length; it is also expected to produce thicker stems, leading to higher diameter/height ratios.

The following tables reports the ecotype effects as well as the standard deviations of family effects within each ecotype and the standard deviation of ecotype effects:

For number of leafs per unit stem length:

ecotype means

glo sp1

0.3447974 0.7508369

family sd within ecotype

glo

[1] 0.05734627

sp1

[1] 0.05666351

ecotype sd

[1] 0.2871133

For D/H ratio:

ecotype means

glo sp1

0.1691919 0.1414167

family sd within ecotype

glo

[1] 0.0127735

sp1

[1] 0.01781857

ecotype sd

[1] 0.01964007

The differences between ecotypes and the dispersion within ecotype are shown by the following box plots:

The differences between ecotypes and the dispersion within ecotype are shown on Suppl. Fig. 7. Allometry is very different between ecotypes, and much less variable within ecotypes, for number of leaves per unit stem length; allometry is also different between ecotypes for D/H, and varies little within *S. globulifera*, but is varies more within *S. sp1*. Notice however that, with diameters in the order of few millimetres, error margins on the estimation of D and D/H may be quite large, and comparatively larger for *S. sp1’s* smaller diameters.

Supp. Fig. 7: Distribution of allometry between number of leaves and height (a) and diameter and height of seedlings belonging each the *Symphonia* ecotypes: *S. globulifera* (glo) and *S. sp1* (sp1).


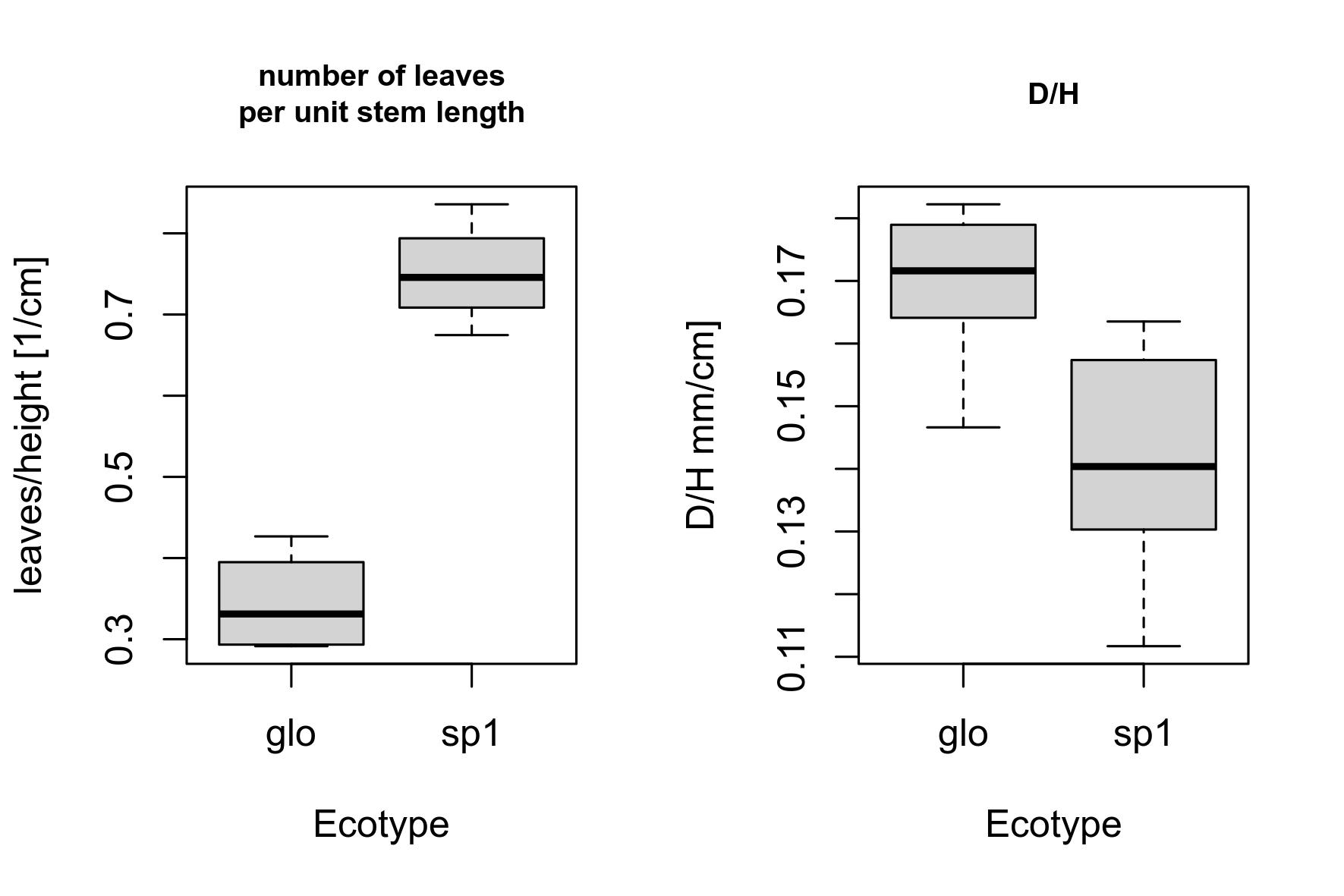

Supplement: Supplementary file 1 — Supplementary Material [file ECE3-10-10735-s001.docx]
